# CNTN1 Aggravates Neuroinflammation and Triggers Cognitive Deficits in Male Mice by Boosting Crosstalk between Microglia and Astrocytes

**DOI:** 10.14336/AD.2023.0228

**Published:** 2023-10-01

**Authors:** Song-Ji Li, Min-Hui Ma, Jun-Mei Li, Xiao-Yu Lu, Cheng-Biao Lu, Shi-Fen Zhou, Lin-Xuan Zhang, Meng-Qing Li, Tong-Ze Shao, Su-Ping Bai, Xiao-Xin Yan, Fang Li, Chang-Qi Li

**Affiliations:** ^1^The International-Joint Lab for Non-invasive Neural Modulation/Key Laboratory for the Brain Research of Henan Province, Xinxiang Medical University, Xinxiang, Henan, China.; ^2^Department of Anatomy and Neurobiology, School of Basic Medical Science, Central South University, Changsha, Hunan, China.; ^3^Department of Human Anatomy and Histoembryology, Xinxiang Medical University, Xinxiang, Henan, China.; ^4^5-year Clinical Medicine Program, Xinxiang Medical University, Xinxiang, Henan, China.; ^5^College of Pharmacy, Xinxiang Medical University, Xinxiang, Henan, China

**Keywords:** CNTN1, cognitive deficits, microglia, inflammation, long-term potentiation

## Abstract

A wealth of knowledge regarding glial cell-mediated neuroinflammation, which contributes to cognitive deficits in Alzheimer’s disease (AD) has emerged in recent years. Contactin 1(CNTN1), a member of the cell adhesion molecule and immunoglobulin supergene family, is centrally involved in axonal growth regulation and is also a key player in inflammation-associated disorders. However, whether CNTN1 plays a role in inflammation-related cognitive deficits and how this process is triggered and orchestrated remain to be fully elucidated. In this study, we examined postmortem brains with AD. CNTN1 immunoreactivity was markedly increased, particularly in the CA3 subregion, as compared with non-AD brains. Furthermore, by applying an adeno-associated virus-based approach to overexpress CNTN1 directly via stereotactic injection in mice, we demonstrated that hippocampal CNTN1 overexpression triggered cognitive deficits detected by novel object-recognition, novel place-recognition and social cognition tests. The mechanisms underlying these cognitive deficits could be attributed to hippocampal microglia and astrocyte activation, which led to aberrant expression of excitatory amino acid transporters (EAAT)1/EAAT2. This resulted in long-term potentiation (LTP) impairment that could be reversed by minocyline, an antibiotic and the best-known inhibitor of microglial activation. Taken together, our results identified *Cntn1* as a susceptibility factor involved in regulating cognitive deficits via functional actions in the hippocampus. This factor correlated with microglial activation and triggered astrocyte activation with abnormal EAAT1/EAAT2 expression and LTP impairment. Overall, these findings may significantly advance our understanding of the pathophysiological mechanisms underlying the risk of neuroinflammation related cognitive deficits.

Alzheimer’s disease (AD), a complicated neuro-degenerative disorder, is characterized by an inflammatory response cascade, which typically leads to progressive cognitive decline and dementia [1-3]. According to the widely accepted amyloid β (Aβ) cascade hypothesis, histopathology involving Aβ aggregation acts as the principal triggering in AD, and induces tau phosphorylation, thus leading to neurodegeneration and AD progression [4-5]. The presence of intracellular neurofibrillary tangles is the prevailing theory for the pathogenic mechanism of AD [5-6]. Furthermore, accumulating evidence has implicated the sustained glia-mediated inflammation cascade and glial-neuron interactions as major contributors to the neuro-degenerative processes and cognitive deficits observed in AD [7-8].

Numerous studies have reported that CNTN1 belongs to the glycosyl phosphatidylinositol-anchored neural cell adhesion molecules of the immunoglobulin superfamily (IgSF) with high expression in the cerebral cortex, corpus striatum, and hippocampus in the rodent brain [9-10]. Previous studies have indicated that CNTN1 regulated synapse formation and axonal tract maturation [11]. In addition, CNTN1 could primarily function as an axonal glycoprotein that is important in facilitating axonal growth and neurites outgrowth [12-14]. CNTN1 was also reported to play an important role in the neuro-inflammatory process involving microglial activation and elevated proinflammatory cytokines in depression [15]. However, the mechanisms and functional roles of CNTN1 as a putative susceptibility factor in neuroinflammation- related cognitive disorders have not yet been characterized.

To gain insight into its role in cognition, CNTN1 overexpression was achieved by bilateral stereotaxical injection into the hippocampus of mice to assess cognitive actions. Similar to the use a battery of behavioral tests to evaluate traits observed in cognitive disorders [16, 17], we evaluated manifestations of cognitive deficits using the novel object recognition test (NORT), novel place recognition test (NPRT), and social recognition test (SRT). In this way, we found that mice with hippocampal CNTN1 overexpression exhibited cognitive deficits. Moreover, we sought to identify the mechanisms by which CNTN1 overexpression in the hippocampus could lead to these cognitive impairments in mice. We integrated multiple approaches such as immuno-histochemical study of postmortem human brains, with stereotactic injection in mice, reverse transcription quantitative polymerase chain reaction, Western blotting, and behavioral pharmacology. Accordingly, we provided causal evidence that over-activation of inflammation by activated microglia and astrocytes contributed to cognitive deficits, and impaired synaptic plasticity upon hippocampal CNTN1 overexpression in mice. In addition, these cognitive deficit-related phenotypes could be pharmacologically reversed by minocycline, which suggested that CNTN1 might be a novel target for therapy of inflammation associated cognitive deficits.

## MATERIALS AND METHODS

### Ethical approval of postmortem brain samples

Postmortem human brains used in the current study were obtained from the Human Brain Bank of Xiangya School of Medicine, which were banked through the voluntary donation program at Xiangya School of Medicine. Brains were banked using a Standardized Operational Protocol established by the China Human Brain Banking Consortium, which included a signed antemortem consent from the donor for donation of body/organs after death for medical education and research [18]. This study was approved by the Ethics Committee of Central South University Xiangya School of Medicine and conducted under standard ethical procedures of the Ethics of the World Medical Association (Declaration of Helsinki). All methods were carried out in accordance with the body/organ donation guidelines and regulations.

### Immunohistochemistry of postmortem brain samples

Sections from postmortem brain samples were processed immunohistochemically with primary antibody, rabbit anti-CNTN1 (1:200, #43782, Signalway Antibody Co, Greenbelt, USA). All free-floating sections were treated with 3% hydrogen peroxide (H_2_ O_2_ ) in phosphate buffer saline (PBS) for 15 min in 5% normal horse serum in PBS containing 0.2% Triton X-100 for 2 h, followed by incubation with the above-mentioned primary antibody at 4- overnight. The sections were then incubated with biotinylated goat anti-rabbit (Jackson Immuno-Research, West Grove, USA) at the concentration of 1:200 for 2 h at room temperature, followed by three washes with PBS. Then sections were incubated with ABC mix (1:200: VECTASTAIN ABC Elite Kit, Vector laboratories, Burlingame, USA) for 2 h at room temperature. Colorization was performed with the immunoreactivity visualized in 3, 3’-diaminobenzidine (DAB), and the reaction was stopped with PBS. After dehydration, sections were mounted on slides with neutral balsam (Sinopharm, Beijing, China). Images were acquired using a Zeiss microscope at 5×, 10× and 20× objectives on a Motic-Olympus microscope equipped with an automated stage and imaging system (Wuhan, China), which could yield a final auto-focused and magnification-adjustable image that covered the entire area of a glass slide.

### Animals and ethics

Adult male wildtype C57BL6/J mice aged 8 weeks were purchased from the Laboratory Animal Center (Central South University, Changsha, China) and were maintained by breeding colonies. Mice were housed in groups 3-5 under controlled environmental conditions (22 ± 1 °C and 55 ± 10 %) with ad libitum access to food and water. Mice were habituated to the experimental environment and handled for 1 week before the experiments started. All behavioral experiments were performed during the light phase from 9:00 am to 5:00 pm and were conducted in accordance with the guidelines of the National Institute of Health Guide for the Care and Use of Laboratory Animals. The study was approved by the Animal Welfare Committee of Central South University.

### Stereotaxic viral injection

All surgeries were performed under stereotaxic guidance (RWD Life Science, Shenzhen, China). Mice were deeply anesthetized with pentobarbital sodium (50mg/kg) before undergoing bilateral craniotomies. Craniotomies were performed using a drill bit with a 0.5 mm diameter, over the hippocampus (2.06 mm posterior to the bregma, ± 0.15 mm lateral to midline, 1.75 mm ventral to the dura), on the basis of the Paxinos and Franklin mouse brain atlas. The mice were injected bilaterally with 500 nL of adeno-associated virus (pAAV_2/9_ -hSyn-CNTN1-CW3SL, titer: 1.0×10^13^ vg/mL, packaged by Obio Technology, Shanghai, China) at a controlled rate of 60 nL/min using a mineral oil-filled glass micropipette with a microsyringe pump (Nanoject III; Drummond, Birmingham, AL, USA), and pAAV-hSyn-MCS-3FLAG-CW3SL (AAV_2/9_, titer: 3.6 × 10^12^ vg/mL, Obio Technology) was used as control virus. The micropipette was maintained in the target site for another 10 min after injection before being slowly withdrawn. Prior to performing behavioral experiments, mice were allowed to recover for at least 3 weeks after injections. Then, mice were handled and habituated to the behavioral environment 1 week before starting the behavioral tests. For all virus injections, mice with incorrect injection sites were excluded from further experiments.

### Open-field test

The open-field test (OFT) apparatus consisted of a 40-cm ×40-cm brightly illuminated square arena that was surrounded by 40-cm-high acrylic walls, equipped with two horizontal planes of 16 infrared photocell-detector pairs placed in the x and y dimensions (RWD, Life Science, China). Mice were individually placed in the center of the arena, and their locomotion activity was monitored for 5 min using an automatic tracking system. Data were analyzed using activity monitoring software. The total distance traveled, and the time spent in the center arena was measured. The chamber was cleaned with 75% ethanol after each use to eliminate olfactory cues between different trials.

### Novel object recognition test

The objects used in this test significantly differed in shape, color, and texture, but not in size or surface complexity. One was a smooth cylindrical plastic object, whereas the other was a rough cuboid glass object. Briefly, each mouse was placed in the center of the arena and was allowed to explore it freely. It was then presented with two identical objects for 5 min during the training phase. Next, the trained mice were returned to their home cage for 1 h. Subsequently, these mice were returned to the same arena used in the training phase, where one object had been replaced with a novel object of a different shape, and mice were allowed to explore freely for another 5 min. The exploration time spent with the familiar and novel objects during the 5 min of the test phase was recorded. The difference in exploration time for a previously familiarized object and for a novel object was determined by calculating individual discrimination ratios (T_Novel_ /(T_Novel_ + T_Familiar_ ) × 100%. The chamber and all objects were cleaned with 75% (v/v) ethanol after each use to eliminate olfactory cues.

### Novel place recognition test

The NPRT protocol consisted of three phases: habituation, training phase and test phase. In the training phase, mice were placed in the open-field arena in which they were presented with two objects that were similar in shape, size, color, and texture in opposite locations and were allowed to explore freely for 5 min. After this period, mice were returned to their home cage for 1 h. One hour later, in the test phase, mice were allowed to explore freely for another 5 min, using the same objects used in the sample phase, but with one of the objects moved to a novel place. The exploration time for the object in the familiar and novel place during the test phase was recorded. Data were expressed as the individual discrimination ratio of the duration of object interaction (T_Novel_ )/(T_Novel_ + T_Familiar_ ) × 100%. Between trials, both objects and the arena were wiped with 75% (v/v) ethanol to avoid any olfactory cues.

### Social recognition test

For the social recognition test, the apparatus was composed of a square open-field box with surrounding grey walls and floor (40×40×40 cm). Two clear cylindrical empty cages (diameter and height of 9 and 10 cm, respectively) were placed in the left and right sides of the apparatus. This test involved three phases: habituation, social interest, and social discrimination. Each mouse was allowed free access to the arena and two empty cages for 5 min during the habituation phase. In the social interest phase, an unfamiliar mouse was introduced to one of the cages, whereas the other cage remained empty. The test mouse was placed into the apparatus again for 5 min to allow familiarization with the stimulus mouse. In the social discrimination phase, an unfamiliar mouse was placed in the other (empty) cage. The test mouse was placed back in the arena and allowed to explore the familiar and unfamiliar mice for 5 min, which was automatically recorded by video. The percentage of time spent exploring the unfamiliar stimulus mouse was measured by estimating individual discrimination ratios (T_unfamiliar_ - T_Familiar_ )/(T_unfamiliar_ + T_Familiar_ ) × 100%.

### Quantitative real-time polymerase chain reaction

The hippocampus tissues were dissected quickly from the mice brain followed by homogenization for extraction of total RNA with cold Trizol reagent according to the manufacturer’s instructions (Invitrogen, Waltham, MA, USA). The cDNA was obtained using HiFiScript cDNA Synthesis Kit (CWBIO Biotech, Changping, China) from the extracted mRNA and Real-time polymerase chain reaction (PCR) was performed using ChamQ Universal SYBR quantitative real-time polymerase chain reaction mastermix (Vazyme Biotech Co., Ltd., Nanjing, China) with the CFX-96 Real-Time PCR System (Bio-Rad, Hercules, CA, USA). Primary data were analyzed by the 2^-ΔΔCt^ method using GAPDH as a housekeeping gene for normalization. The primer sequences used for the amplification of *Cntn1* were as follows: (mice, NM_ 001159647.1) *Cntn1*: 5′-CGCGTTTCAAGTCAAAGTG A-3′; 5′-TTTGACCCCTACCTCTGTGG-3′. The primer sequences used for the amplification of inflammation-related genes were listed in Table 1.

**Table 1:** List of PCR primers.

Gene	Primer sequences (5’-3’)	Gene accession No.
IL1α	*F:* GTTCCTGACTTGTTTGAAGACC R: GTTGGACATCTTTGACGTTTCA	NM_010554.4
IL6	*F:* CTCCCAACAGACCTGTCTATAC *R*: CCATTGCACAACTCTTTTCTCA	NM_031168.2
Ccl2	*F:* TTTTTGTCACCAAGCTCAAGAG *R*: TTCTGATCTCATTTGGTTCCGA	NM_011333.3
CD11b	*F:* GAGCATCAATAGCCAGCCTCAGTG *R*: CCAACAGCCAGGTCCATCAAGC	NM_001082960.1
CD68	*F:* GAAATGTCACAGTTCACACCAG *R*: GGATCTTGGACTAGTAGCAGTG	NM_001291058.1
GAPDH	*F:* ACTTTGGCATTGTGGAAGGG *R*: AGTGGATGCAGGGATGATGT	NM_001289726.1

### Western blot analysis

The hippocampal tissue was homogenized in tissue lysis buffer with a proteinase inhibitor cocktail (CWBIO), followed by centrifugation at 12000 rpm at 4°C for 20 min. The supernatant was harvested and denatured by boiling in 6 × sodium dodecyl sulfate (SDS) loading buffer for 8 min. Then, equal amounts of proteins were loaded onto 10% SDS-polyacrylamide gels, electro-phoretically separated, and transferred to nitrocellulose membranes. Then, membrane were blocked with 5% skim milk for 2 h at room temperature, followed by overnight incubation with the following primary antibodies: CNTN1 (Signalway Antibody Co, 43782, 1:1000), Vimentin (Abcam, Cambridge, MA, USA, ab92547, 1:1000), GFAP (Boster Bio, Pleasanton, CA, USA, BM0055, 1:1000), NR2A (Boster Bio, PA1058-1,1:1000), NR2B (Proteintech, Rosemont, IL, USA, 21,920-1-AP, 1:1000), GluR1 (Bioss, Woburn, MA, USA, bs-10042R, 1:1000), GluR2 (Boster Bio, BM5334, 1:1000), PSD95 (Proteintech, 20,665-1-AP, 1:1000), Synaptophysin (Proteintech, 17,785-1-AP, 1:1000), EAAT1 (Abcam, ab181036, 1:1000), EAAT2 (Abcam, ab205248, 1:1000) and GAPDH (Proteintech, 60,004-1-Ig, 1:1000) at 4-, and followed by 2 h incubation with horseradish peroxidase-conjugated secondary antibodies for goat anti-rabbit (Jackson Immuno-Research,111-035-003, 1:2000) or goat anti-mouse (Jackson Immuno-Research,115-035-003,1:2000). Finally, blots were exposed to enhanced chemiluminescent substrate (CWBIO). The densitometric value of immunoblots was measured with Image J software (NIH).

### Immunohistochemistry

Mice were deeply euthanized under pentobarbital sodium (50 mg/kg) anesthesia by transcardial perfusion with saline followed by 4% paraformaldehyde before brain removal. Whole brains were removed and equilibrated in 15% and 30% sucrose for at least 48 h before sectioning. Fresh frozen brains were sectioned at 30-µm thickness using a cryostat (Leica, Wetzlar, Germany; CM1850). Immunohistochemical staining was performed on free-floating sections washed in PBS three times. Next, endogenous peroxidases were blocked by incubating the sections in PBS with 3% H_2_ O_2_ for 15 min followed by washing in PBS three times. Then, 5% bovine serum albumin in PBS containing 0.2% Triton X-100 was used to block nonspecific staining. The sections were incubated with goat anti-Iba1 primary antibody (Abcam, ab5076, 1:1000) or mouse anti-NeuN (Millipore, Billerica, MA, USA, MAB377, 1:500) at 4 °C overnight followed by three washes with PBS. Sections were then incubated for 2 h at room temperature with rabbit anti-goat (Proteintech, SA00004-4, 1:200) or goat anti-mouse (Jackson Immuno-Research, 115-065-003, 1:200) IgG peroxidase-polymer secondary antibody and then followed by three washes with PBS. Colorization was performed using the avidin-biotin complex (ABC) kit (VECTASTAIN ABC Elite Kit, Vector laboratories, USA) for 2 h at room temperature, followed by the application of 3-3′-diaminobenzidine (DAB, Santa Cruz Biotechnology, Dallas, TX, USA). Controls involved replacing the primary antibody with Norma goat serum (1:1000) for Iba1 immunostaining or Norma mouse serum (1:1000) for NeuN immunostaining, exhibited no staining. Sections were mounted on gelatin-coated glass slides, dried and dehydrated in a graded series of alcohol (75%, 95% twice and 100% twice) followed by xylene before coverslipping with neutral balsam (Sinopharm). Images were acquired using a Zeiss microscope (Carl Zeiss, Oberkochen, Germany) with 10, 20× and 40× objectives.

### Nissl staining

For Nissl (cresyl violet) staining, the brain sections were mounted on gelatin-coated slides and allowed to dry overnight. Before Nissl staining, the sections (30 μm-thick) were degreased in xylene and in an ethanol gradient and were then rinsed with distilled H_2_ O. The sections were stained with 0.1% cresyl violet (Boster Bio, China) for 30 min, rinsed in distilled H_2_ O, dehydrated in a graded series of ethanol, immersed in xylene, mounted with neutral balsam (Sinopharm), and cover-slipped. Finally, sections were observed under a microscope (Leica) to evaluate the hippocampal neuronal loss and perform further image analysis.

### Hematoxylin-eosin staining

For hematoxylin-eosin (HE) staining, the brain sections were mounted on gelatin-coated slides and were allowed to dry overnight. Before HE staining, the sections (30 μm-thick) were degreased in xylene and a gradient of ethanol and then rinsed with dH_2_ O. The sections were then stained with hematoxylin (Boster Biotech, China) for 15 min, counterstained with eosin for 3 min, and then rinsed in distilled H_2_ O. The stained sections were processed sequentially with 75% alcohol, 95% alcohol, absolute ethanol, and xylene and mounted with neutral balsam (Sinopharm) for further microscopic observation (Leica) and image analysis.

### Image analysis

Microglial cell numbers and immune-reactive areas were determined using Image-Pro Plus software (IPP, version 6.0, Media Cybernetics, Inc, Bethesda, MD, USA). Briefly, high-resolution images were randomly captured in CA1 of the hippocampus using 5×, 20 × and 40 × objectives. Ionized calcium-binding adapter molecule 1 (Iba-1) labeled cells were automatically counted and divided by the area to calculate the cell density. For each animal, at least one section of the hippocampus was analyzed. The immunoreactive areas in each region were thresholded, divided by the area, and represented as percentages. To examine microglial morphology in the hippocampus, the ramifications of at least three microglia were analyzed with a Sholl analysis plugin in Fiji software (Image J &Fiji, National Institutes of Health, Bethesda, MD, USA). First, we used the line segment tool to draw a line from the somatic center to the longest process of each microglia. Second, we set the first shell at 3-μm and subsequent shells in 5-μm steps to determine the number of intersections at each Sholl radius. For quantification analysis of NeuN, Nissl and Hematoxylin staining, three or four random non-overlapping areas of the hippocampus were analysed for each animal of the different groups by Image J. All images were recorded using the same parameters between groups and analyzed blindly by several observers.

### Minocycline administration

Minocycline (50 mg/kg; Med Chem Express, Monmouth Junction, USA) was diluted in sterile saline at a constant volume (10 ml/kg) and administered intraperitoneally for 4 consecutive days. The dose of minocycline selected to inhibit microglia was on the basis of previously described with modification [19]. Control mice received an equal volume of sterile saline.

### Preparation of brain slices

Mice were anesthetized with isoflurane and transcardially perfused with ice-cold cutting solution (in mM: 225 sucrose, 3 KCl, 6 MgCl_2_, 1.25 NaH_2_ PO_4_, 24 NaHCO_3_, 0.5 CaCl_2_, 10 glucose, pH 7.3 adjusted with HCl, osmolarity 300-310 mOsm/kg) and subsequently for rapid decapitation. The perfused brain was rapidly removed and the 350-μm horizontal slices were prepared in ice-cold cutting solution with a Leica VT1200S vibratome (Leica) and then incubated under constant oxygenation with 95% O_2_ and 5% CO_2_. Then, slices containing the dorsal hippocampus were allowed to recover under constant oxygenated artificial cerebrospinal fluid (aCSF in mM: 126 NaCl, 3 KCl, 1.25 NaH_2_ PO_4_, 2 MgSO_4_, 24 NaHCO_3_, 2 CaCl_2_, and 10 glucose) for 30 min at 32 °C before equilibrating at room temperature for at least an additional hour for subsequent recording.

### Electrophysiological recording

After 1 h of equilibration at room temperature, brain slices were transferred to an interface-type recording chamber where they were perfused with aCSF of 32 °C, at a rate of 4 mL/min, with their surface exposed to warm, humidified carbogen (95% O_2_ -5% CO_2_ ). Field extracellular recordings were performed by stimulating the Schaeffer collateral fibers through a bipolar twisted 50-μm nickel/chromium electrode and recording in the CA1 stratum radiatum with a borosilicate glass pipette filled with aCSF (resistance was 3-5 MΩ). Input-output (I/O) curves were obtained for each slice, and stimulus intensity for all subsequent recordings was set to elicit a field excitatory postsynaptic potential (fEPSP) slope that was 30-40 % of the maximum response. After a stable baseline (15-30 min) was established, long-term potentiation (LTP) was induced by a single high-frequency stimulation (HFS) train (100 Hz, 1 s at standard intensity). Responses were recorded for 1 h after tetanic stimulation and measured as an fEPSP slope expressed as a percentage of baseline.

### Statistical analysis

Data analyses were conducted using GraphPad Prism 8.02 (GraphPad Software, Inc., La Jolla, USA). All data were expressed as mean ± standard error of the mean (SEM). Before statistical analysis, the D'Agostino & Pearson test was used to test the normality of distribution. The comparisons between two groups were evaluated by two-tailed Student’s t-test for normally distributed variables and a one-way or two- way ANOVA was used to compare values between more than two groups, followed by post hoc Bonferroni tests for multiple comparisons. The non-parametric Kruskal-Wallis test was used to make comparison with non-parametric datasets (non-normal distribution or n<6) among more than two groups. *P* values of less than 0.05 were considered statistically significant.

**Table 2:** Demographic information and clinical profiles of subjects used in this study.

Case	Group	Age(y)	Sex	Clinical diagnosis or cause of death	Postmortem delay (h)	Braak NFT stage	Thal Aβ phase
1	Control	76	M	Heart stroke	7	0	0
2	Control	78	M	heart failure	6	I	0
3	Control	80	M	Iron-deficiency anemia	7	II	0
4	Control	77	M	Multisystem failure	12	III	0
5	Control	81	F	Chronic renal failure	7	IV	0
6	Control	81	M	Cardiovascular failure	6	II	0
7	Control	78	F	Ovary cancer	4.5	II	0
8	Control	70	F	Pneumonia	12	II	0
9	Aged/AD	89	M	Coronary heart	8	III	1
10	Aged/AD	72	M	Alzheimer’s disease (AD)	8	V	4
11	Aged/AD	76	M	Colon cancer	5	VI	C
12	Aged/AD	74	M	Multisystem failure	5	V	5
13	Aged/AD	80	M	Alzheimer’s disease (AD)	22	VI	5
14	Aged/AD	76	M	Chronic Heart Disease	5	VI	C
15	Aged/AD	83	F	Multisystem failure	12	III	4
16	Aged/AD	85	M	Astrocytoma	12	III	4

## RESULTS

### Increased CNTN1 expression in postmortem human AD brains

**Figure 1.**
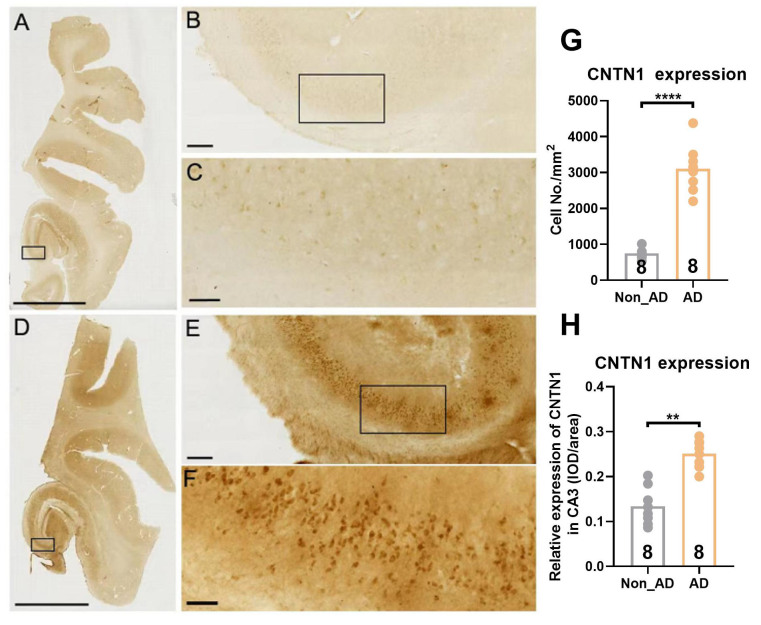
CNTN1 expression was increased in the hippocampus of AD postmortem brain relative to non-AD control. (A-F) Representative images of CNTN1 expression in hippocampal subregions at different magnifications in non-AD controls (A-C) and AD postmortem brains (D-F). Immunohistochemistry (IHC) was done on frozen sections of OCT embedded hippocampus. (G) Quantitative analysis of CNTN1 expression in hippocampus by number of non-AD control and AD postmortem brain. (H) Quantitative analysis of CNTN1 expression in hippocampus by integrated optical density (IOD) of non-AD control and AD postmortem brain. Data were analyzed by unpaired two-tailed Student’s *t* test. Data were presented as mean ± sem. **p < 0.01, compared with control group. Scale Bar: A&D: 8 mm; B&E: 300 μm; C&F: 60 μm.

To evaluate whether AD was associated with increased CNTN1 expression, we performed immunohistochemical staining in the hippocampus of eight patients with AD and eight non-AD individuals of similar ages and sex, as listed in Table 2. Student’s unpaired t-test showed a significant increase in CNTN1 expression in sections from postmortem AD brains in comparison with those from non-AD brains. This included the number and integrated optical density (IOD) of neurons positive for CNTN1 immunoreactivity (Fig.1A-H, t=9.752, *p*<0.0001 for number; t=6.368, *p*=0.007 for IOD). Thus, our findings supported the morphological evidence that, in AD brains, CNTN1 expression was significantly upregulated as compared to that in non-AD brains.

### Local overexpression of CNTN1 within the hippocampus triggered cognitive deficits in mice

**Figure 2.**
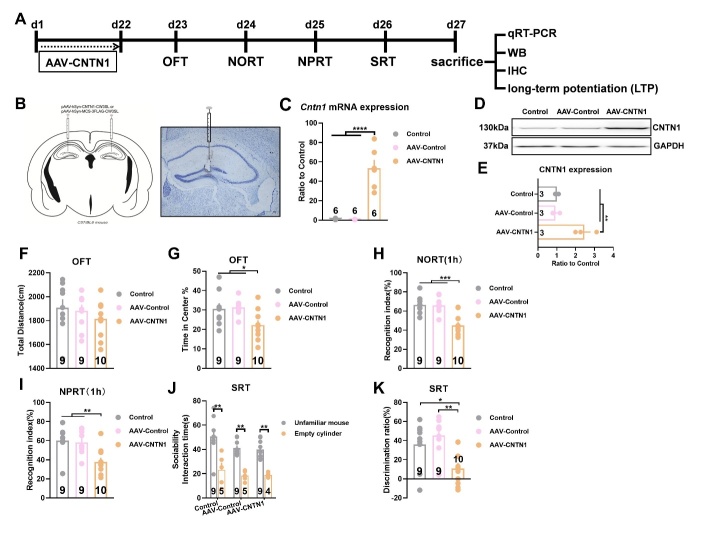
CNTN1 overexpression by AAV stereotactic injection in hippocampus triggered cognitive deficits in mice. (A) Schematic illustration of behavior paradigm for assessment of cognitive behavior after AAV-CNTN1virus injection into the dorsal hippocampus in mice. (B) Left panel showed schematic illustration of AAV-Cntn1virus injection into the dorsal hippocampus in mice. The right panel showed Nissl staining of virus injection into the dorsal hippocampus in mice. (C) Quantitative real time qPCR analysis of *Cntn1* mRNA expression after AAV-CNTN1 or AAV-Control injection in hippocampus in mice. (D&E) Representative immunoblot (D) and quantitative analysis (E) of CNTN1 expression after AAV-CNTN1 or AAV-Control injection in hippocampus in mice. (F&G) The effect of AAV-CNTN1 or AAV-Control overexpression in hippocampus on Locomotion (F) and anxiety like behaviors (G) in mice as manifested by open field test (OFT) test. (H-K) The cognitive deficits with manifestation by several behavioral traits in different groups, including novel object recognition task (NORT (H)), novel place recognition test (NPRT (I)) and social recognition test (SRT(J&K)). Data were analyzed by one-way ANOVA followed by Bonferroni’s multiple comparison tests except for panel J, which was analyzed by unpaired two-tailed Student’s t test. Data were presented as mean ± sem.*p < 0.05, **p < 0.01 and ***p < 0.001.

Considering that CNTN1 expression was markedly increased in postmortem AD brains, we evaluated whether hippocampal CNTN1 overexpression led to development of cognitive deficits in mice. A schematic diagram of cognitive behavior is shown in Fig. 2A. Somatic gene transfer with a stereotaxically-injected recombinant adeno-associated virus (AAV) vector induced CNTN1 overexpression in the dorsal hippocampus of mice. Of note, the bilateral stereotactic injection of AAV-CNTN1 virus into the hippocampus markedly augmented the *Cntn1* mRNA and CNTN1 protein expression in comparison with those in mice injected with AAV-Control (Fig. 2B-2E, F_2,15_ =37.77, *P*<0.0001 for mRNA; F_2,6_ =21.01, *P*=0.002 for protein). As shown in Fig. 2F, no significant differences were observed in the total distance traveled in the OFT between the AAV-CNTN1 and AAV-Control mice. Primarily, hippocampal CNTN1 overexpression triggered anxiety-like behavioral traits, with less time spent in the central arena in the OFT test by mice injected with AAV-CNTN1 than by the AAV-Control mice (Fig. 2G, F_2,25_ =4.915, *P*=0.0158). Behaviorally, cognitive performance could be evaluated using a battery of behavioral tests, including the NORT, NPRT and SRT. Using the NORT, there was no spontaneous preference for objects during the learning phase, as the percentage of time spent on each object was similar. During the recognition phase, hippocampal CNTN1 overexpression significantly impaired recognition performance during the NORT, which was proven to be a failure to discriminate between familiar and novel objects, as evidenced by less time spent exploring the novel object. The AAV-control and control mice could clearly distinguish the novel object, which was reflected by the recognition index (Fig. 2H, F_2,25_ =15.59, *P*<0.0001). Using the NPRT, the AAV-CNTN1 mice showed no preference for objects in either location. Nevertheless, AAV-Control and control mice showed preferential exploration of an object located in a novel place, as compared with a familiar place, as shown by the recognition index (Fig. 2I, F_2,25_ =7.915, *P*=0.0022). Furthermore, the SRT was used to evaluate cognitive performance. The results of sociability interaction time in mice showed spontaneous preference for animate, rather than abiotic objects (Fig. 2J). The discrimination results showed a significantly reduced discrimination ratio in the mice with AAV-CNTN1 compared with the AAV-Control and control mice (Fig. 2K, F_2,25_ =7.476, *P*=0.0029). Taken together, these results preliminarily supported the notion that increased CNTN1 expression may be a cellular basis for cognitive deficits in mice.

### CNTN1 overexpression induced cognitive deficits with microglia and astrocyte activation

**Figure 3.**
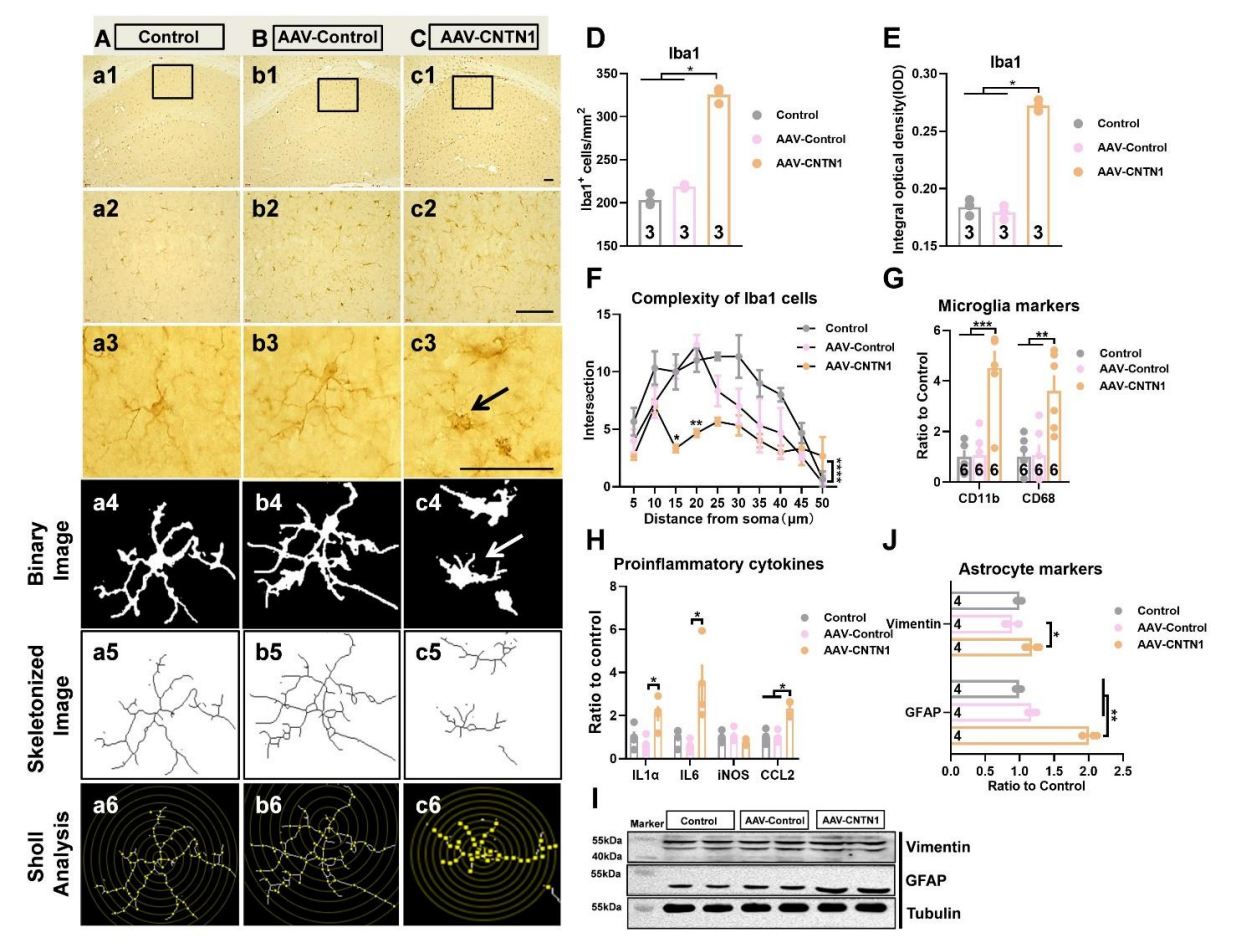
CNTN1 overexpression by AAV stereotactic injection activated microglia and astrocyte in the hippocampus. (A-C) Representative images showed immunostaining against microglial marker Iba1 on brain sections in the hippocampus of mice in different groups. (D&E) Quantiative analysis of Iba1 positive microglia of hippocampus in different groups by total number of microglia (D) and integrated optical density (IOD) (E). (F) Quantitative analysis complexity of Iba1 positive microglia in hippocampus with Sholl analysis in different groups. (G) Quantitative real time qPCR detection of mRNA expression levels of CD11b and CD68 in hippocampus in different groups. (H) Quantitative real time qPCR detection of mRNA expression levels of IL1α, IL6, iNOS and CCL2 in hippocampus in different groups. n=4 per group. (I&J) Representative immunoblot (I) and quantitative analysis (J) of vimentin and GFAP expression in hippocampus in different groups. Data in Fig. 3D, E, H, J were analyzed by Kruskal-Wallis statistical test; Data in Fig. 3F was analyzed by two-way ANOVA followed by Bonferroni’s multiple comparison test. Data in Fig. 3G was analyzed by one-way ANOVA followed by Bonferroni’s multiple comparison tests. Data were presented as mean ± sem. *p < 0.05, **p < 0.01 and ****p < 0.0001. Scale bar: 50 μm.

CNTN1 belonged to the IgSF and played a pivotal role in inducing neuroinflammation [12, 15]. Therefore, the inflammation-related cellular mechanisms through which hippocampal CNTN1 overexpression resulted in cognitive deficits were assessed. As shown in Fig. 3A-C, the representative images of Iba-1-positive cells exhibited ameboid microglia that demonstrated a series of morphological characteristics. These characteristics included shorter and thicker processes, enlarged cell bodies with retraction of complex branches, in the mice injected with AAV-CNTN1 (Fig. 3A-C). In addition, hippocampal CNTN1 overexpression led to a significant elevation of Iba1 immunostaining intensity by quantitative analysis of number and IOD, as compared to those in their control counterparts (Fig. 3D&E, Kruskal-Wallis statistical test (KWS)=7.2,*P=*0.0036 for number; KWS=5.804,*P=*0.0321 for IOD). To quantify the extent of process retraction induced by CNTN1 overexpression, binary and Sholl analyses were performed. Of note, the results showed significantly decreased microglial branches in the hippocampus of the mice with AAV-CNTN1 and presented an ameboid-like shape as compared with the AAV-Control and control mice (Fig. 3F, F_2,60_ =31.7, *P*<0.0001). Additionally, the expression levels of CD11b and CD68 were significantly elevated in the hippocampus of the AAV-CNTN1 mice (Fig. 3G, F_2,15_ =19.6, *P*<0.0001 for CD11b; F_2,15_ =9.969, *P*=0.0018 for CD68). Considering that microglia could be activated after CNTN1 overexpression, we investigated the molecular actors related to neuroinflammation. Importantly, the results revealed elevated the expression of interleukin (IL)1α and IL6 in the mice with AAV-CNTN1 compared with the AAV-control and control mice (Fig. 3H, KWS=6.962, *P*=0.0194 for IL1α; KWS =8.346, *P*=0.0024 for IL6). The expression of other proinflammatory cytokines expression were also significantly increased, such as chemokine ligand (CCL2) (Fig. 3H, KWS=7.385, *P*=0.0145 for CCL2), but without any significant differences in iNOS expression. Furthermore, representative immunoblots, and quantitative analysis indicated significantly increased expression of vimentin and glial fibrillary acidic protein (GFAP) in the mice with AAV-CNTN1 (Fig. 3I and J, KWS=6.731, *P*=0.0215 for vimentin; KWS=9.846, *P*=0.0002 for GFAP). Taken together, these findings provide evidence that CNTN1 overexpression might cause both microglia and astrocyte activation and may trigger inflammatory responses in the hippocampus.

### CNTN1 overexpression induced long-term potentiation impairment through aberrant excitatory amino acid transporters 1 and 2 expression

**Figure 4.**
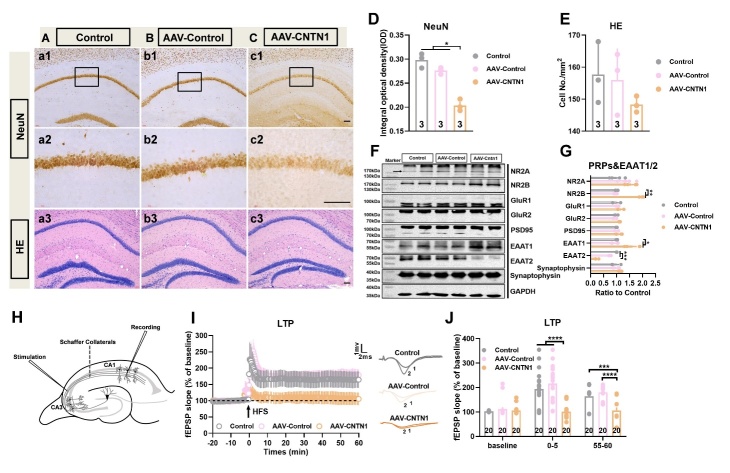
Hippocampal CNTN1 overexpression induced impairment of LTP at Schaffer collateral-CA1 synapses. (A-C) Representative images showed immunostaining against neuronal marker NeuN at low magnification of brain sections in hippocampus of mice in different groups (a1-c1. (A-C&D) Representative images showed immunostaining against neuronal marker NeuN (a2-c2) and quantitative analysis at high magnification of brain sections (D) in hippocampus of mice in different groups. (A-C&E) Representative images showed HE staining (a3-c3) and quantitative analysis on brain sections (E) in hippocampus of mice in different groups. (F&G) Representative immunoblots (F) and quantitative analyses of PRPs, EAAT1 and EAAT2 expression (G) in hippocampus in different groups. n=4 per group. (H) Schematic position of the stimulus electrode and recording electrode in the dorsal hippocampal slices in mice. Recording electrode positioned in CA1 apical dendrites was performed by stimulating electrodes to stimulate schaeffer collateral inputs. (J&K) Representative images (I) and Quantitative analysis (J) showed the time-course of the fEPSP slope recorded from the hippocampal Schaeffer collateral-CA1 (Sch/CA1) synapses in different groups. The traces adjacent to the panel were the field EPSPs at the times indicated on the panel in different groups. Trace1 showed pre HFS, Trace 2 showed post HFS. Data in Fig. 4D, E and G were analyzed by Kruskal-Wallis statistical test; Data in Fig. 4J were analyzed by one-way ANOVA followed by Bonferroni’s multiple comparison tests. Data were presented as mean ± sem. *p < 0.05, **p < 0.01,***p < 0.001 and ****p < 0.0001. Scale bar: 50 μm.

To evaluate the possible impact of CNTN1 overexpression on hippocampal neurons, immunostaining for NeuN as a mature neuronal marker was performed in the hippocampus. The results showed markedly decreased NeuN immunoreactivity in CA1 in the mice injected with AAV-CNTN1 in comparison to mice injected with AAV-Control and in control mice (Fig. Aa1, Cc2 and D, KWS=6.489, *P=*0.0107). Additionally, the NeuN immunoreactivity of the dentate gyrus was also decreased in the AAV-CNTN1 mice, as compared to AAV-Control and control mice (data not shown), however, no significant loss of cells was observed with hematoxylin and eosin staining (Fig. 4Aa3, Cc3 and E).

Furthermore, representative immunoblots, and quantitative analysis showed prominently increased expression of EAAT1, but decreased expression of EAAT2 in AAV-CNTN1 mice, as compared to the AAV-Control and control mice (Fig. 4F and G, F_2,9_ =14.79, KWS=7.423, *P*=0.0132 for EAAT1; KWS=8.846, *P*=0.0002 for EAAT2). This aberrant expression of EAAT1/ EAAT2 led to over-activation of NR2B, which was involved in regulation of glutamate signaling [20, 21] (Fig. 4F and G, KWS=8.0, *P*=0.0048). Nevertheless, no significant differences in the expression of NR2A, GluR1, GluR2, PSD95 or synaptophysin were observed (Fig. 4F and G). To evaluate whether synaptic plasticity was affected by hippocampal CNTN1 overexpression, electrophysiological recordings were used to assess LTP. To achieve this, slices received HFS of the Schaeffer collateral fibers while the amplitude of the postsynaptic potential of pyramidal neurons in the CA1 was recorded (Fig. 4H). In hippocampal slices, HFS reliably induced a robust and reproducible LTP in hippocampal slices from the AAV-Control and control mice. Bonferroni post-hoc analysis showed a significant LTP magnitude decrease in slices from AAV-CNTN1 mice as compared with that in AAV-Control and control mice (Fig. 4I and J, F_2,57_ =18.65, *P*<0.0001 for 0-5 min; F_2,57_ =15.94, *P*<0.0001 for 55-60 min). Taken together, these results might preliminarily prove that hippocampal CNTN1 overexpression could lead to aberrant expression of EAAT1/EAAT2, thus resulting in LTP impairment in the hippocampus.

### Minocyline ameliorated cognitive deficits induced by CNTN1 overexpression through inhibition of microglia and astrocytic activation

**Figure 5.**
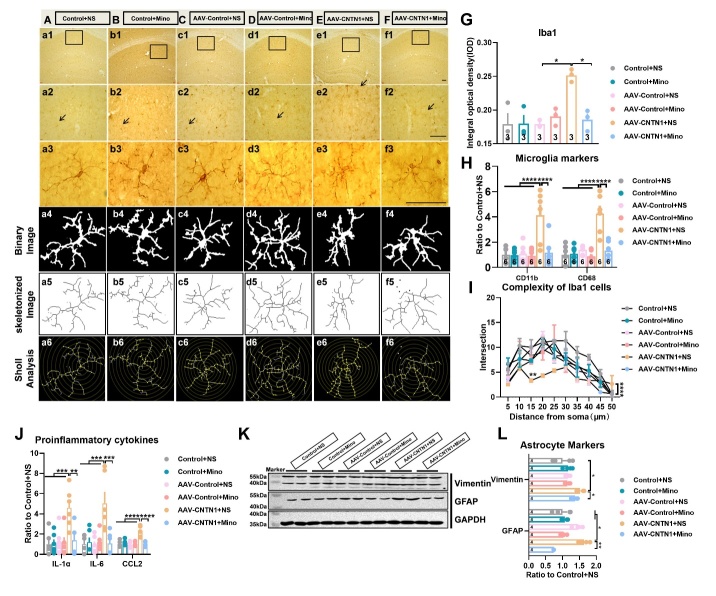
Minocycline inhibited microglia activation and the following astrocyte activation induced by hippocampal CNTN1 overexpression. (A-F) Representative images showed immunostaining against microglial marker Iba1 on brain sections in hippocampus of mice in different groups. (G) Quantiative analysis of Iba1 positive microglia in hippocampus of mice in different groups by integrated optical density (IOD). (H) Quantitative real time qPCR detection of mRNA expression of CD11b and CD68 in hippocampus. (I) Quantitative analyses of complexity with Iba1 positive microglia in hippocampus in different groups. (J) Quantitative real time qPCR detection of mRNA expression of IL1α, IL6 and CCL2 in hippocampus in different groups. n=6 per group. (K&L) Representative immunoblots (K) and quantitative analyses (L) of vimentin and GFAP expression in hippocampus in different groups. Data in Fig. 5G, and L were analyzed by Kruskal-Wallis statistical test; Data in Fig 5. I was analyzed by two-way ANOVA followed by Bonferroni’s multiple comparison tests. Data in Fig. 5H, I and J were analyzed by one-way ANOVA followed by Bonferroni’s multiple comparison tests. Data were presented as mean ± sem. *p < 0.05, **p < 0.01,***p < 0.001 and ****p < 0.0001. NS: Saline. Scale bar: 50 μm.

**Figure 6.**
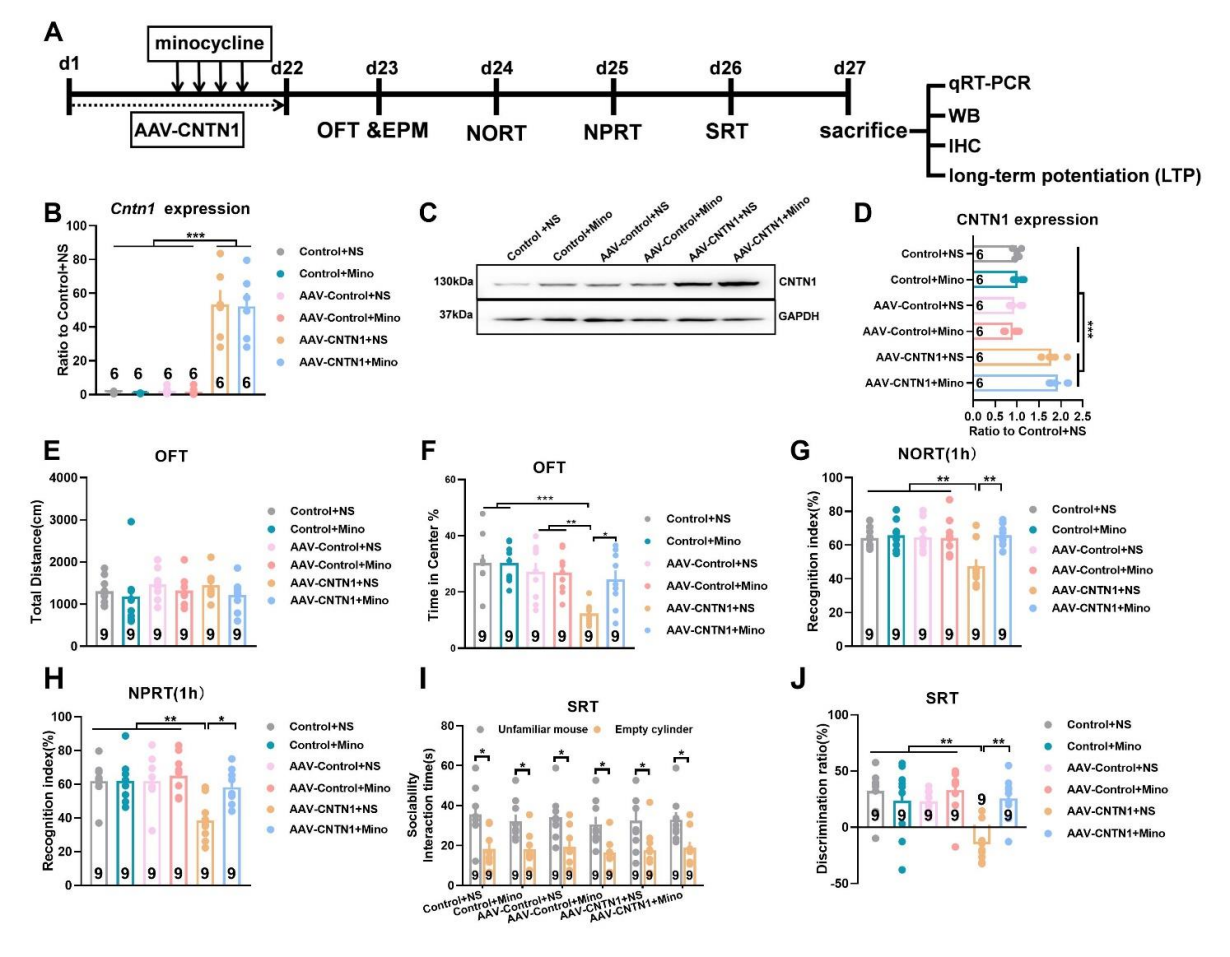
Microglia inhibition by minocycline ameliorated cognitive deficits in mice with CNTN1 overexpression in hippocampus. (A) Schematic diagram of behavior paradigm of cognitive deficits in mice. (B) Quantitative real time qPCR detection of *Cntn1* mRNA expression in hippocampus in different groups. (C&D) Representative immunoblots (C) and quantitative analyses (D) of CNTN1 expression in hippocampus in different groups. (E&F) The total distance traveled (a measurement index of locomotor activity) and anxiety like behaviors in different groups in the OFT test. (G-J) The cognitive deficits with manifestation by several behavioral traits in different groups, including NORT (G), NPRT (H) and SRT (I&J). Data were analyzed by one-way ANOVA followed by Bonferroni’s multiple comparison tests except for Fig. 6I, which was analyzed by unpaired two-tailed Student’s t test. Data were presented as mean ± sem. ^*^p < 0.05, ^**^p < 0.01 and ^***^p < 0.001.

To elucidate whether activated microglia would play a role in the cognitive deficits induced by hippocampal CNTN1 overexpression in mice, we used minocyline, a drug commonly used to inhibit microglial activation. As expected, minocyline markedly reversed the effects on microglial morphology alteration and immunoreactivity of Iba1 positive cells in the hippocampus, as compared with saline (Fig. 5A-F and G, KWS=13.17, *P=*0.0217) and also had an impact on microglial markers, including CD11b and CD68 (Fig. 5H, F_5, 92_ =8.183, *P*<0.0001 for CD11b; F_5,92_ =13.48, *P*<0.0001 for CD68). Quantitative Sholl analysis indicated that minocycline led to a considerable recovery in the process complexity of microglia, with morphological changes similar to those observed in the control mice (Fig. 5I, F_5,120_ =12.32, *P*<0.0001), accompanied by a reversal in the levels of pro-inflammatory cytokines IL1α, IL6 and CCL2 (Fig. 5J, F_5,12_ =5.167, *P*=0.0093 for IL1α; F_5,18_ =7.105, *P*=0.0008 for IL6, F_5,30_ =16.95, *P*<0.0001 for CCL2). Microglial inactivation by minocycline markedly decreased astrocyte activity, as evidenced by the expression of vimentin and GFAP, which are markers for reactive astrocytes (Fig. 5K and L, KWS=14.30, *P*=0.0138 for vimentin; KWS=18.37, *P*=0.0025 for GFAP). A behavioral schematic diagram is illustrated in Fig. 6A. Notably, CNTN1 overexpression in the hippocampus showed prominently augmented its *Cntn1* mRNA (Fig. 6B, F_5,30_ =30.63, *P<*0.0001) and CNTN1 protein expression (Fig. 6C and D, F_2,6_ =21.01, *P*=0.002), which was not reversed by minocycline. As shown in Fig. 6E, no statistically significant differences were found in locomotor activity between the minocycline and saline groups (Fig. 6E). The time spent in the central arena during the OFT used to reflect the anxiety-like phenotype was significantly decreased by CNTN1 overexpression and this behavior was alleviated by minocycline (Fig. 6F, F_5,48_ =6.26, *P*=0.0002). More importantly, minocycline remarkably increased the percentage of time spent exploring the novel object to a value similar to that in the control+NS mice (Fig. 6G, F_5,48_ =5.406, *P*=0.0005). Similar results were observed in the NPRT, with increased time spent exploring the object localized in a novel location (Fig. 6H, F_5,48_ =5.714, *P=*0.0003). In addition, minocycline reversed the cognitive decline detected by the SRT, with an increased discrimination ratio (Fig. 6J, F_5,48_ =6.608, *P<*0.0001), without affecting spontaneous preference for animate objects (Fig. 6I). These results might suggest that minocycline could ameliorate cognitive deficits in mice with CNTN1 overexpression, indicating that activated microglia might act as a cellular basis for the observed cognitive deficits.

### Minocyline restored LTP impairment induced by CNTN1 overexpression in mice

**Figure 7.**
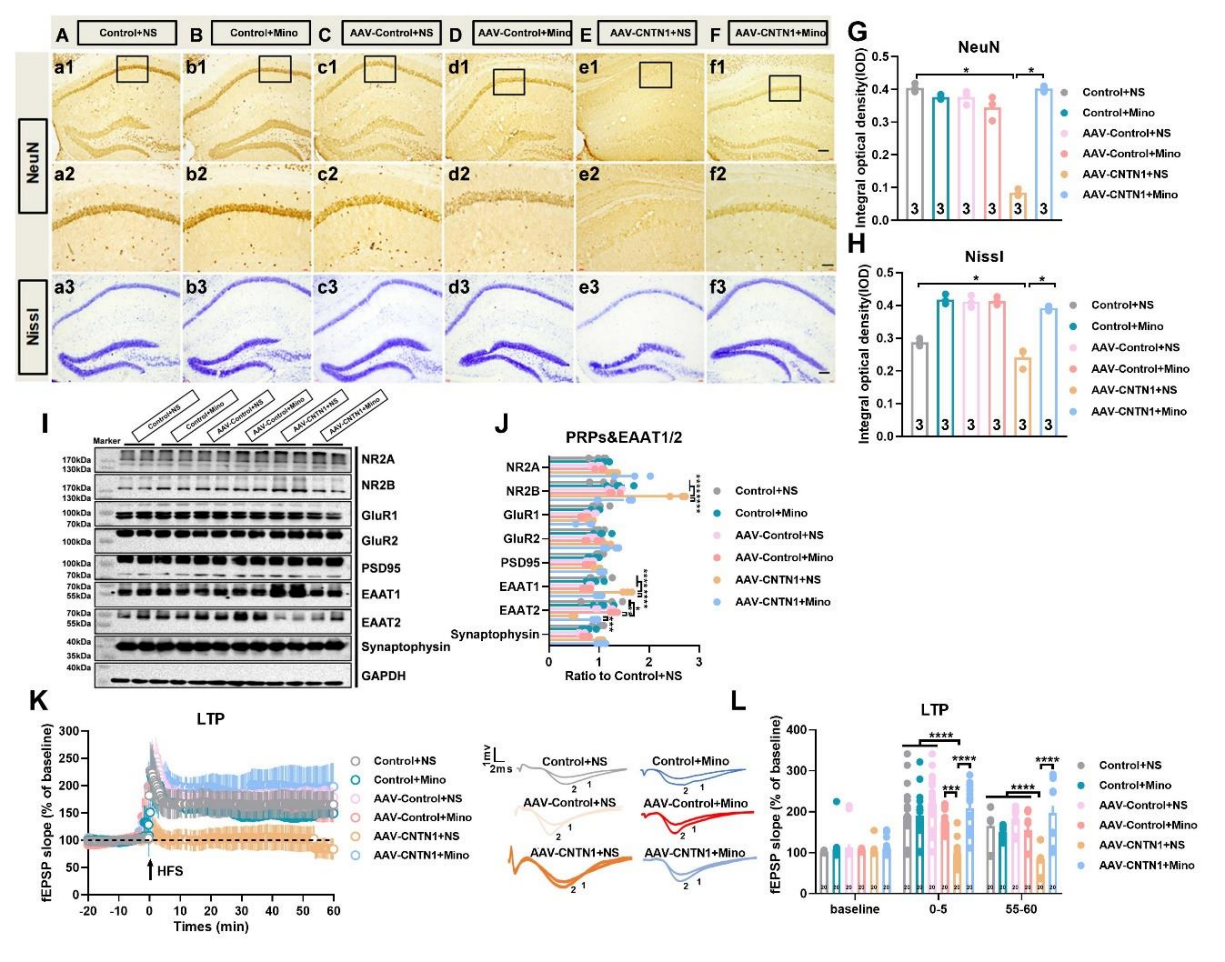
Minocycline mitigated impairment of LTP induced by CNTN1 overexpression in hippocampus. (A-F) Representative images showed NeuN expression in hippocampus at low magnification in different groups (a1-f1). (A-F&G) Representative images (a2-f2) and quantitative analysis of NeuN expression (G) at high magnification of CA1 subregion in different groups. (A-F&H) Hippocampal Nissl staining (a3-f3) and quantitative analysis (H) in different groups. (I&J) Representative immunoblots (I) and quantitative analyses (J) of PRPs and EAATs expression in hippocampus in different groups. n=4 per group. (K) Representative images showed the time-course of the fEPSP slope recorded from the hippocampal Schaeffer collateral-CA1 (Sch/CA1) synapses in different groups. The traces adjacent to the panel were the field EPSPs at the times indicated on the panel in different groups. Trace1 showed pre HFS, Trace 2 showed post HFS. Data in Fig. 7G, and H were analyzed by Kruskal-Wallis statistical test; Data in Fig.7J, K and L were analyzed by one-way ANOVA followed by Bonferroni’s multiple comparison tests. Data were presented as mean ± sem.^*^p < 0.05, ^***^p < 0.001 and ^****^p < 0.0001. Scale bar: 50 μm

As illustrated in Fig. 7G, minocycline remarkably abrogated the CNTN1 overexpression induced decrease in NeuN immunoreactivity in the CA1 region of the hippocampus (Fig.7A-F and G, KWS=13.82, *P=*0.0138). Furthermore, representative images showed decreased Nissl staining following CNTN1 overexpression, which was also reversed by minocycline (Fig.7H, KWS=14.19, *P=*0.0144). Additionally, Fig. 7I and J show that minocycline could significantly reverse the aberrant expression of EAAT1 and EAAT2 to the control levels (Fig. 7I and J, F_5,18_ =25.50, *P*<0.0001 for EAAT1; F_5,18_ =7.183, *P*=0.0007 for EAAT2), which was accompanied by significant suppression of the increase NR2B expression induced by hippocampal CNTN1 overexpression (Fig. 7I&J, F_5,18_ =25.74, *P*<0.0001). The electrophysiological results revealed that minocycline notably restored LTP impairment in AAV-CNTN1 mice to levels similar to those in the control condition, as compared with saline treatment (Fig. 7K&L, F_5,114_ =13.76, *P*<0.0001 for 0-5 min; F_5,114_ =15.7, *P*<0.0001 for 55-60 min). Altogether, these results indicated that microglial activation played an important role in the hippocampal synaptic plasticity when CNTN1 overexpression induced neuroinflammation.

## DISCUSSION

In this study, we provided experimental based evidence indicating that hippocampal CNTN1 overexpression mediated neuroinflammation and cognitive dysfunction in mice. Specifically, we showed that CNTN1 expression was largely increased in human postmortem AD brains as compared with non-AD brains. Additionally, hippocampal CNTN1 overexpression appeared to cause cognitive deficits in mice, which coincided with the activation of microglia and astrocytes, leading to abnormal EAAT1/ EAAT2 expression with the blockade of synaptic LTP in the hippocampus, which could be prevented by minocycline. These results advance our understanding of the functional significance of increased CNTN1 expression in patients with AD.

Previous studies have reported that CNTN1 belongs to the IgSF of adhesion molecules. It plays various roles, including those in neural development and neurodegenerative disorders that are associated with inflammation, and is widely expressed in the central nervous system [9, 10, 15]. The hippocampus is a structure considered to be of crucial importance in terms of cognitive deficits [22-24]. In this study, the role of CNTN1 in cognitive deficits was demonstrated by the finding that hippocampal CNTN1 overexpression caused cognitive impairment in mice, as detected by a series of behavioral assays, including the NORT, NPRT and SRT. Evidence of memory impairment was observed in the NORT performed 1 h after training in AAV-CNTN1 mice, in which they spent less time exploring a novel object. CNTN1 overexpression could alter the function of the hippocampus through several mechanisms, including an inflammatory cascade and neurogenesis [15]. The NPRT assessed the ability to distinguish a configuration previously learnt from that in which an object was situated in a novel place. Our results demonstrated that AAV-CNTN1 mice were unable to distinguish between the object in the new place and that in the familiar place. In the SRT, AAV-CNTN1 mice also showed a decreased discrimination ratio, which was also observed in previous studies related to another adhesion molecule was involved [25].

It would therefore be valuable to understand the precise mechanisms underlying the cognitive impairment induced by CNTN1 overexpression. Next, we therefore assessed changes in microglial markers and found that CNTN1 overexpression markedly increased CD11b and CD68 expression associated with the inflammatory response, accompanied by amoebic microglia with morphological alterations, characterized by the retraction of numerous fine processes and enlarged cell bodies, as previously reported [15, 26]. Moreover, our results also showed that astrocytes were activated with manifestation of increased vimentin and GFAP expression due to hippocampal CNTN1 overexpression, which predominantly expressed large amounts of EAATs, which are critical for maintaining low glutamate concentrations [27-29]. Inflammatory cytokines secreted by microglia could cause astrocyte activation and mediate communication between microglia and astrocyte [30-32]. In a recent study, the author has shown that microglial polarization could govern the communication between microglia and astrocytes [33, 34]. This prompted us to identify the specific mechanisms by which these two glial cells crosstalk with respect to CNTN1 overexpression, since this was not determined in our previous study. Inflammatory factors and amyloid β-induced microglial polarization promoted inflammatory crosstalk with astrocytes. We further showed abnormal expression of EAAT1 and EAAT2, which was due to the activation of astrocytes by proinflammatory cytokines released from microglia activated by CNTN1 overexpression. The regulation of glutamate homeostasis by astrocytes is controlled by both glutamate transporters and glutamine synthetase, which function as enzymes that convert glutamate to glutamine. Whether CNTN1 overexpression regulates glutamine synthetase expression was not addressed in this study. Furthermore, as an inhibitor of activated microglia, minocycline also inhibited astrocytes as evidenced by changes in vimentin and GFAP expression, which proved the existence of crosstalk between microglia and astrocytes. However, the precise mechanisms involved require further research.

N-methyl-d-aspartate (NMDA) receptors, including NR2B, play crucial roles in the induction of LTP in the hippocampus. Nevertheless, excess glutamate in the synaptic clefts resulted in prolonged over-activation of glutamate receptors. This leads to calcium overload, which results in neuronal vulnerability and impairment of LTP through multiple mechanisms. In our study, we showed that CNTN1 overexpression in hippocampus resulted in activation of NR2B, which seems to contradict the notion that decreased NR2B was related to the impaired LTP. This finding may be attributed to damage to LTP caused by the inflammatory response. In addition, the up-regulation of NR2B may have been involved in the activation of NR2B due to excessive glutamate.

It is widely reported that microglia-associated neuroinflammation is a crucial regulator of synaptic plasticity and cognitive performance in the hippocampus [35, 36]. Hence, synaptic plasticity was probably hampered by the increased microglia and inflammatory response in the hippocampus, as evidenced by morphological and molecular changes AAV-CNTN1 mice. Thus, we revealed hippocampal synaptic plasticity and the results indicated that HFS promoted an increase in the neural population spike slope of evoked responses that responded to the Schaeffer collateral fibers of CA1 in the AAV-Control and control mice. However, AAV-CNTN1 mice showed diminished efficiency in inducing LTP compared with the control mice, which was reversed by minocycline, a centrally penetrant tetracycline antibiotic to inhibit microglial activation.

The present study has some limitations. The mechanism by which CNTN1 affects microglial activation needs to be elucidated. It was reported that CNTN1 could function in various signaling pathways, such as phosphatidylinositol 3-kinase (PI3K)/protein kinase B (AKT) and the Notch signaling pathway, which are involved in microglia activation mediated inflammation [37-38]. Therefore, further work is required to elucidate the detailed mechanisms underlying microglial activation and the subsequent astrocyte activation. Furthermore, since hippocampal CNTN1 overexpression induced both microglia and the astrocyte activation, since microglia and astrocytes could regulate each other reciprocally as reported, the present study only focused on the effects of activated microglia acting as the initiating cells after CNTN1 overexpression. Whether activated astrocytes act as the driving cells upon CNTN1 overexpression requires further assessment. To address neuronal synaptic changes, electrophysiological field recordings were performed only for hippocampal LTP. Since fEPSP represents the neuronal activity of the hippocampus, whether CNTN1 also regulates other electrophysiological parameters such as miniature and spontaneous neurotransmitter transmission, the NMDA/AMPA ratio after CNTN1 overexpression are required for future investigation.

Despite several limitations, the present study uncovered the detrimental effects of hippocampal CNTN1 overexpression on cognitive and synaptic function. The possibility of neuroinflammation related negative effects of CNTN1 should be considered, as major efforts have focused on developing CNTN1-based therapeutic targets to mitigate neuroinflammation-associated cognitive deficits.

## Data Availability

All materials and data of this research are available from the corresponding authors upon reasonable request.
